# Supplementary material for: A large-strain and ultrahigh energy density dielectric elastomer for fast moving soft robot
Source: Nat Commun. 2024 May 18;15:4222. doi: 10.1038/s41467-024-48243-y (PMC11102557; doi:10.1038/s41467-024-48243-y)
Supplement: Supplementary file 1 — Supplementary Information [file 41467_2024_48243_MOESM1_ESM.pdf]

Supplementary information

A large-strain and ultrahigh energy density dielectric elastomer for fast moving soft robot

Wenwen Feng[1], Lin Sun[1], Zhekai Jin[1], Lili Chen[1], Yuncong Liu[1], Hao Xu[1], Chao Wang[1,*]

[1]Key Lab of Organic Optoelectronics & Molecular Engineering, Department of Chemistry, Tsinghua University, Beijing 100084, China

*Corresponding author. E-mail: chaowangthu@mail.tsinghua.edu.cn

This file includes:

Supplementary Figures 1-34 (Pages 1-34)

Supplementary Tables 1-2 (Pages 35-36)

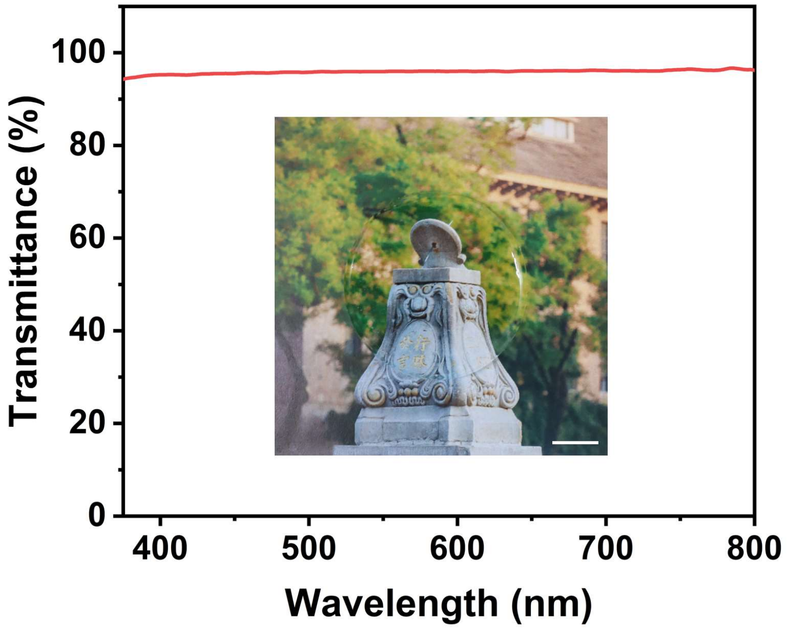

Supplementary Fig. 1. Transmittance spectrum of the PFED10 film. An average transmittance of 95% was recorded in the visible range of 400 to 800 nm. Scale bar, 1 cm.

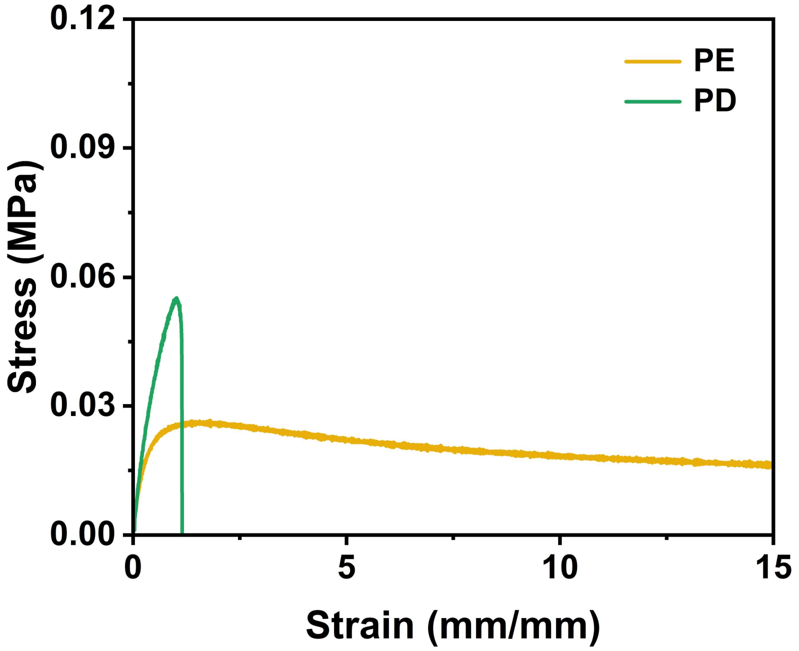

Supplementary Fig. 2. Stress-strain curves of poly(2-ethylhexyl acrylate) (PE) and poly(dodecyl acrylate) (PD). According to the stress-strain curves, the mechanical property of PE is soft and weak. The mechanical property of PD is soft but brittle. The low stretchability of PD comes from the aggregation of the long side chains serving as physical crosslinkers.

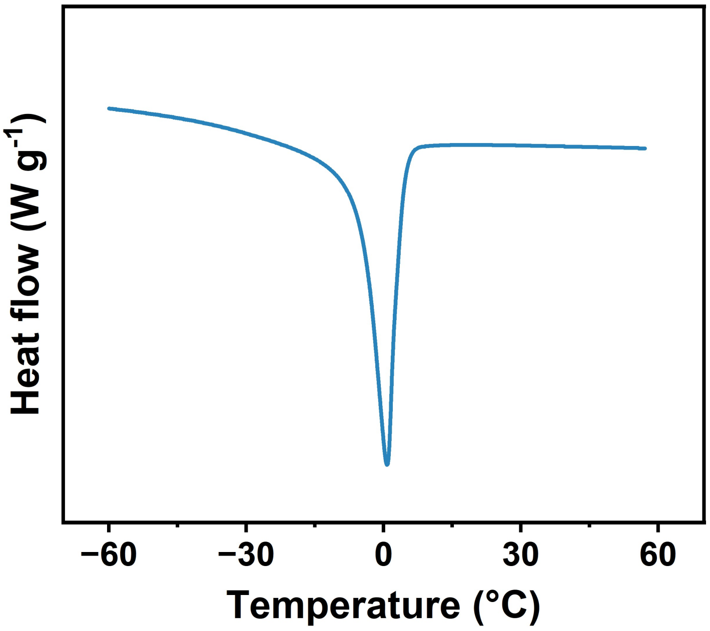

Supplementary Fig. 3. DSC curve of PD. The large and obvious melting peak of PD indicates that PD has a certain crystallinity, which is achieved by the packing of long side chains.

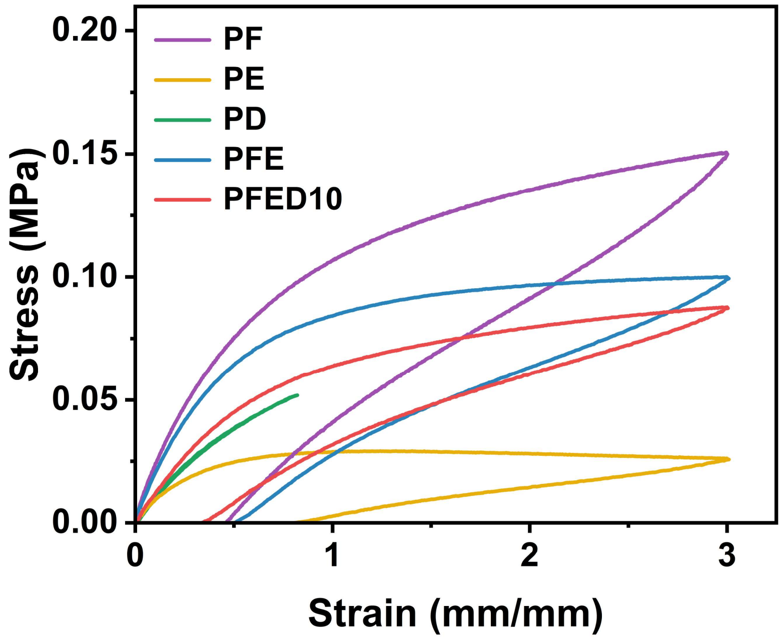

Supplementary Fig. 4. Cyclic Stress-strain curves of PF, PE, PD, PFE, and PFED10. According to the cyclic stress-strain curves, PF and PE show poor elasticity. However, PD shows excellent elasticity, indicating that the aggregation of the long side chain serves as physical crosslinkers. Compared with PFE, the addition of DA in PFED10 improves the elasticity of elastomer.

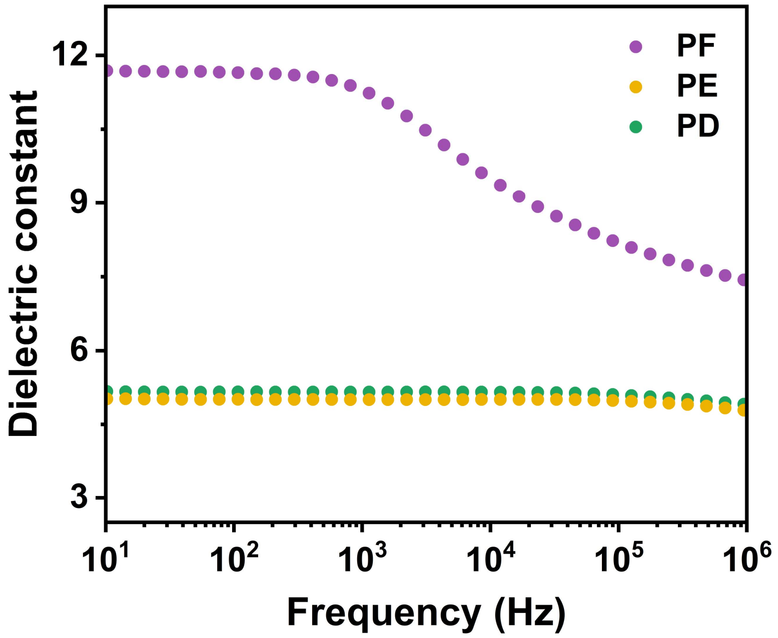

Supplementary Fig. 5. Dielectric constants of PF, PE, and PD from 10 Hz to 10^6 Hz. Due to the rich and highly polar CF_3 groups, PF exhibits a high dielectric constant. Compared with PF, PE and PD have lower dielectric constants.

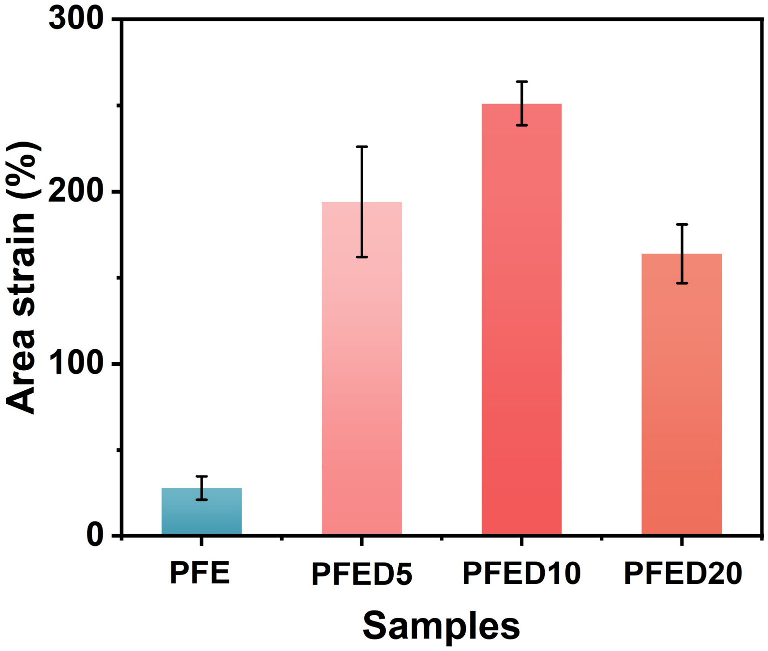

Supplementary Fig. 6. The maximum actuation area strain of PFE, PFED5, PFED10, and PFED20. Because the stress-stain curve of PFE showed obvious yield behavior, the electromechanical property of PFE is too poor to achieve a large actuation area strain. The maximum actuation area strain of PFE is only 28%. Compared with PFE, the series of PFED copolymers achieve large actuation area strains (> 150%) by introducing DA to improve their electromechanical properties. Error bars show s.d., $n = 3$.

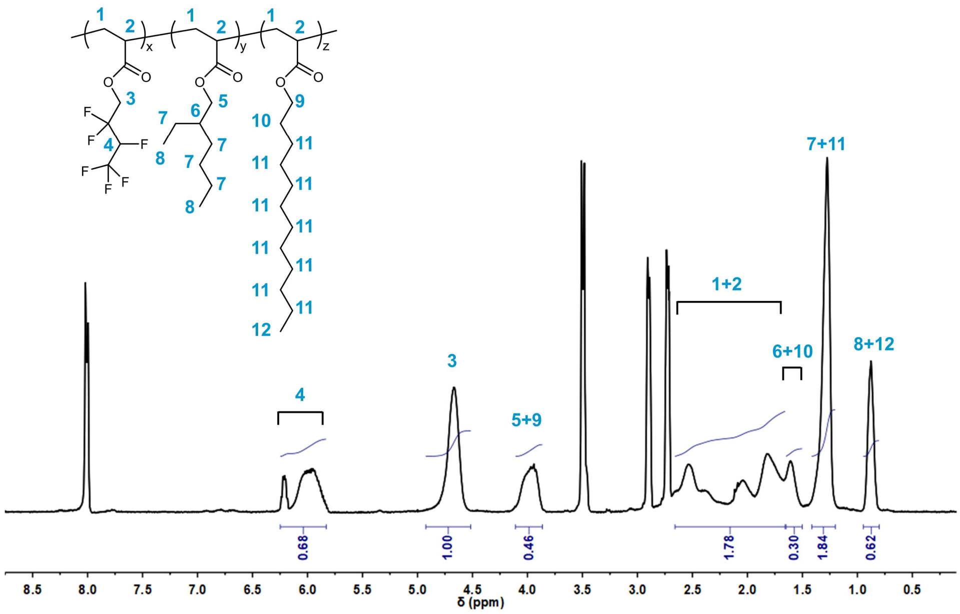

Supplementary Fig. 7. ^{1}H NMR (DMF-d7) spectrum of PFED10. The chemical shift of the vinyl disappeared.

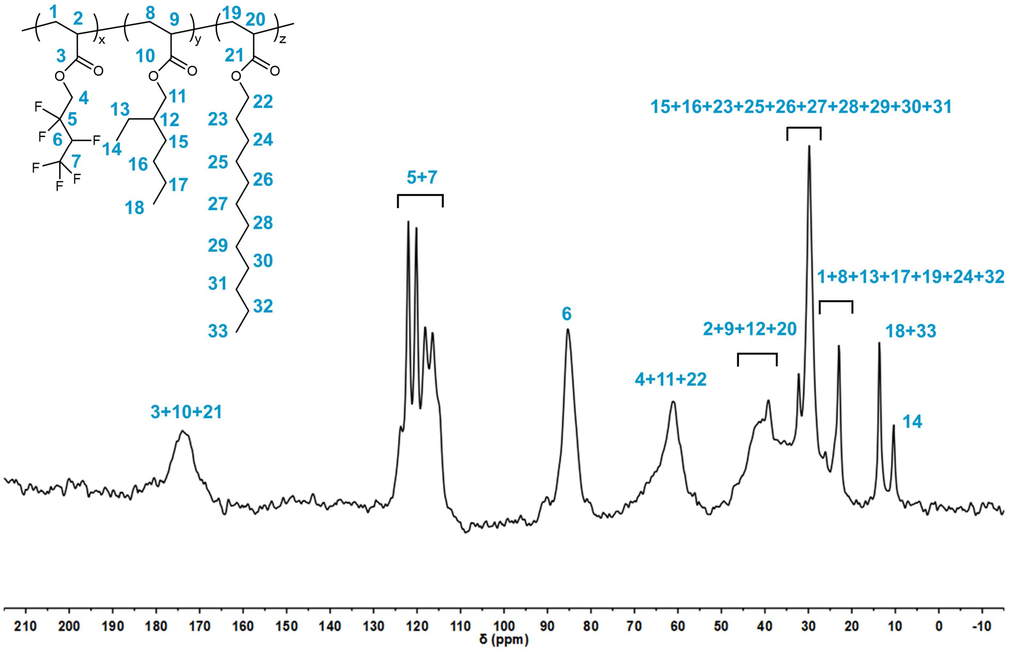

Supplementary Fig. 8. Solid-state ^{13}C NMR spectrum of PFED10. The chemical shift of the carbon-carbon double bond disappeared.

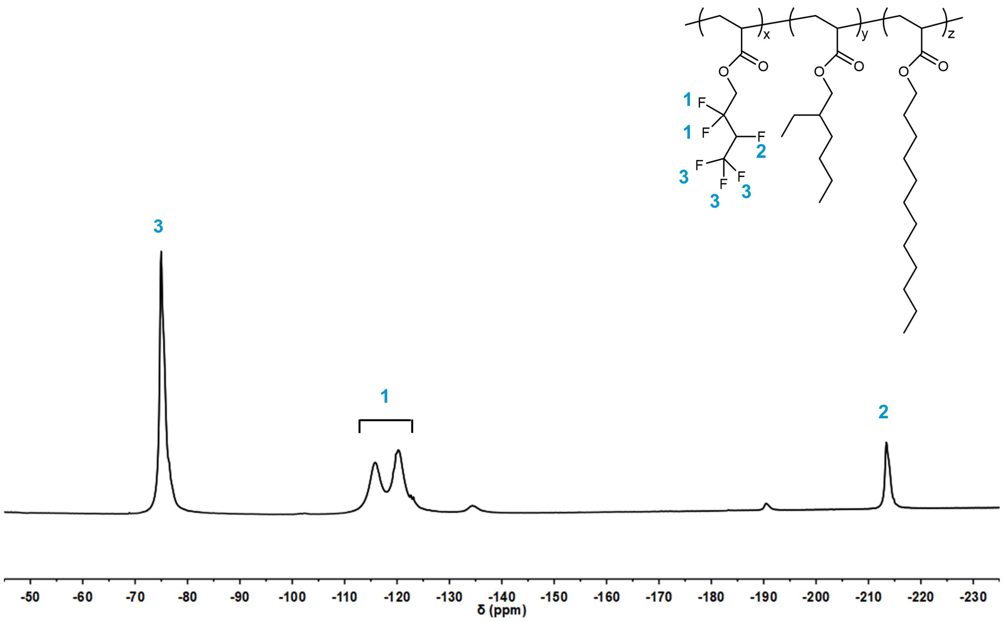

Supplementary Fig. 9. Solid-state ^{19}F NMR spectrum of PFED10.

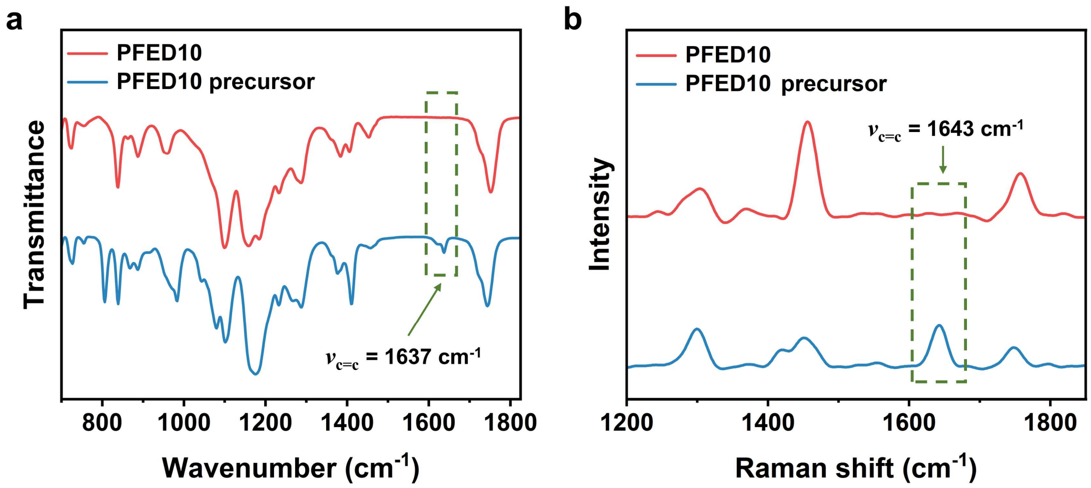

Supplementary Fig. 10. FTIR and Raman spectra of PFED10 and PFED10 precursor. **a**, FTIR spectra of PFED10 and PFED10 precursor. **b**, Raman spectra of PFED10 and PFED10 precursor. The PFED10 precursor was a mixture of HFBA, EA, and DA with a molar ratio of 100: 10: 10. The FTIR spectra of the precursor of PFED10 before and after polymerization showed that the absorption peak corresponding to the *v*(C=C) (from acrylate) at 1637 cm^{-1} vanished, indicating that the conversion of the comonomers was almost 100%. In Raman spectra, the Raman band of C=C bond at 1643 cm^{-1} in monomers vanished in the corresponding copolymer indicating the full conversion of the comonomers.

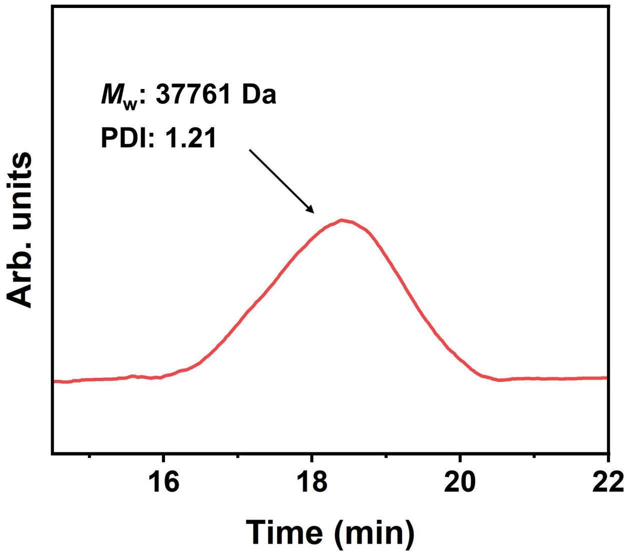

Supplementary Fig. 11. GPC curve of PFED10.

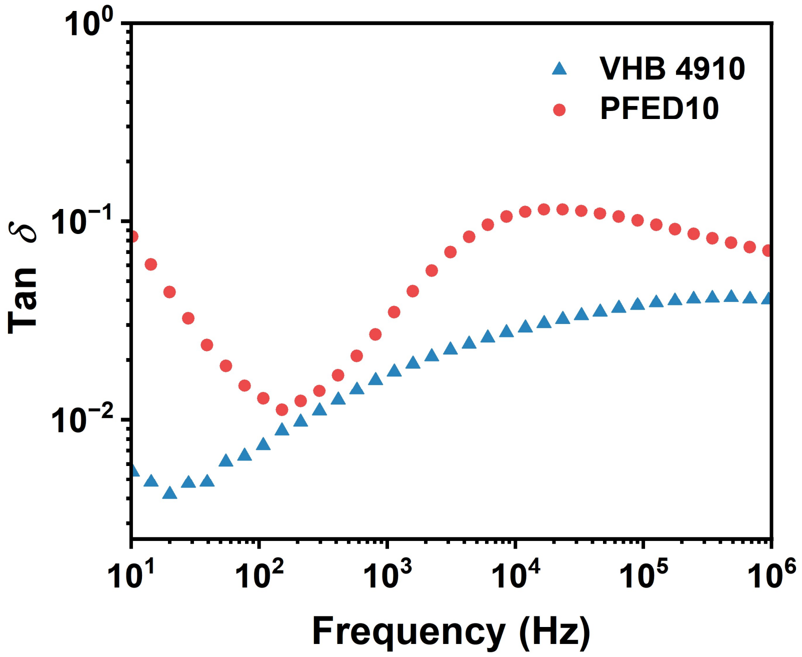

Supplementary Fig. 12. Dielectric loss factors (tan δ) of VHB 4910 and PFED10 in the range of 10 Hz to 10^6 Hz.

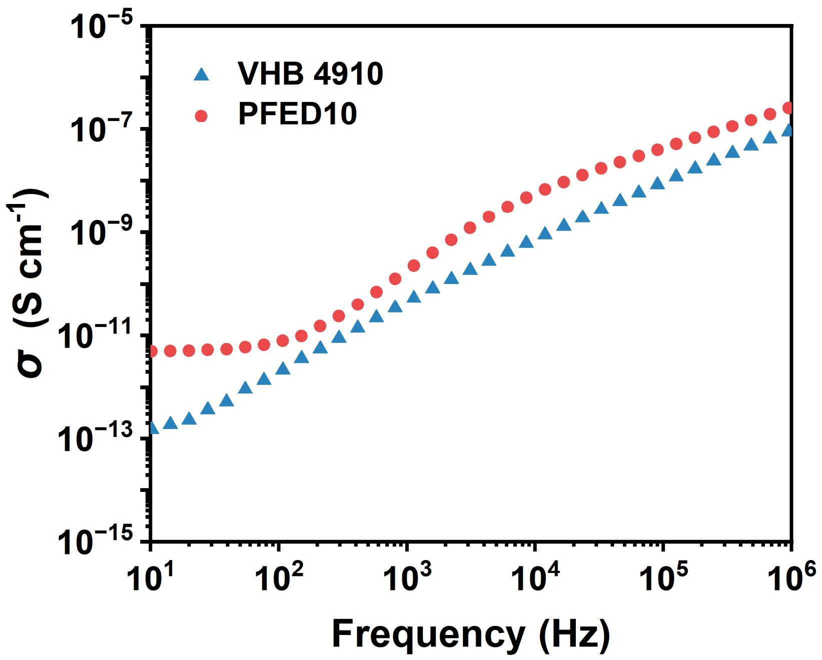

Supplementary Fig. 13. Conductivity of VHB 4910 and PFED10 in the range of 10 Hz to 10^6 Hz.

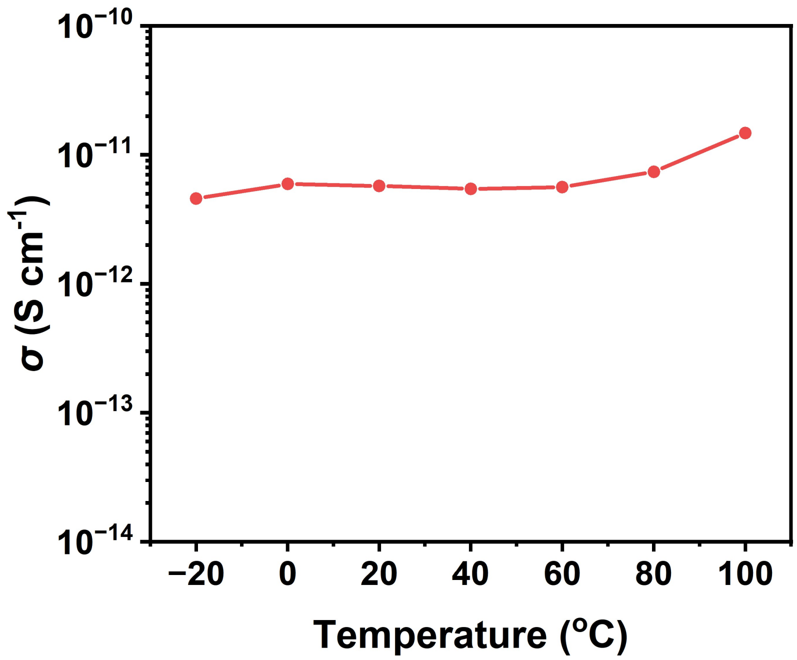

Supplementary Fig. 14. Conductivity of PFED10 from -20 °C to 100 °C.

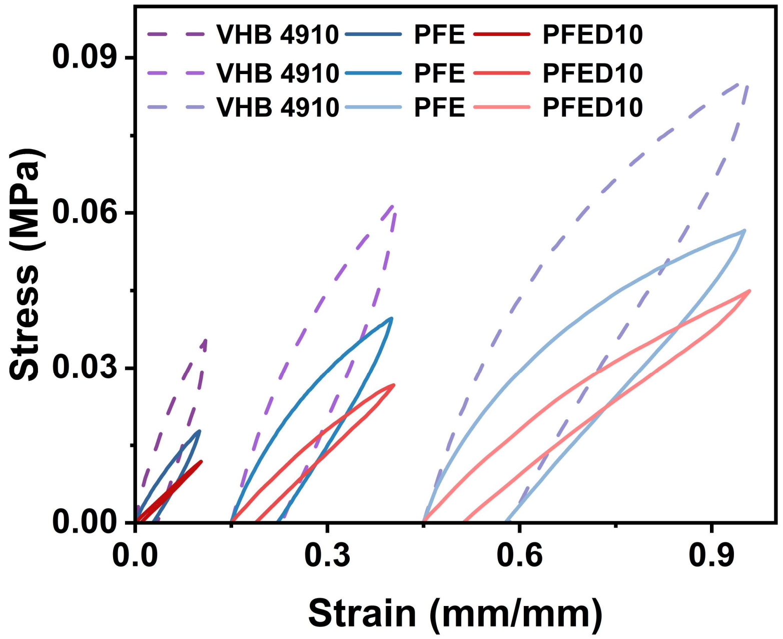

Supplementary Fig. 15. Cyclic stress-strain curves of VHB 4910, PFE, and PFED10 subjected to strains from 10 to 50%. Due to the introduction of DA, PFED10 exhibits excellent elasticity compared to PFE and VHB 4910.

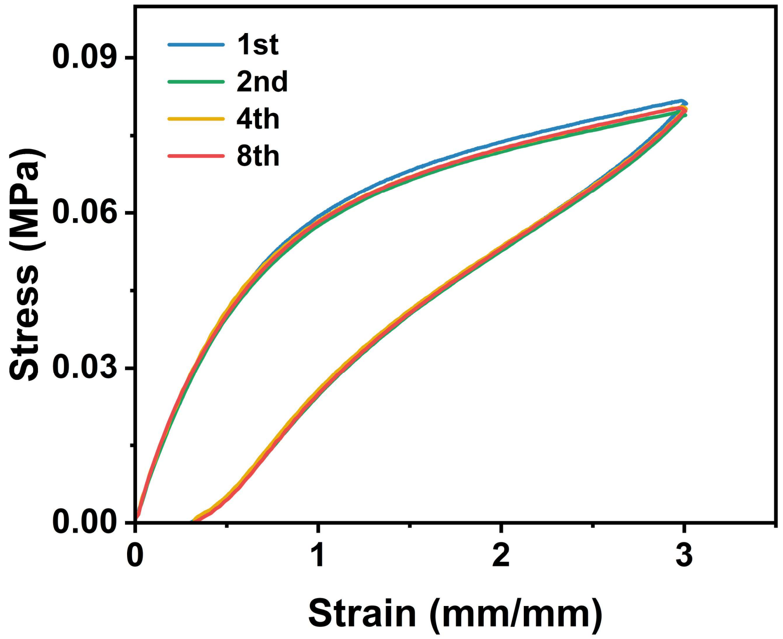

Supplementary Fig. 16. Cyclic stress-strain curves of PFED10 under 300% strain for 8 cycles. The interval of each cycle was 1 min.

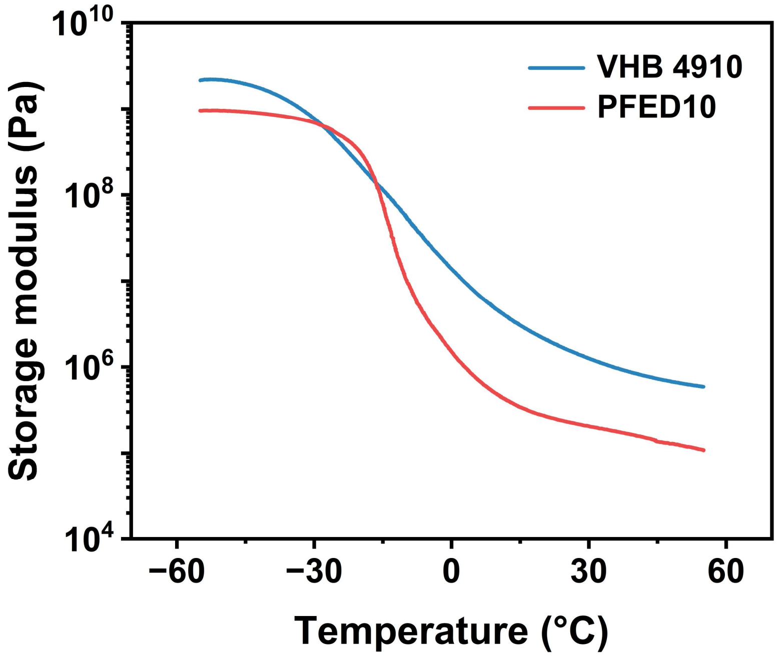

Supplementary Fig. 17. Storage modulus of VHB 4910 and PFED10 from -55 °C to 55 °C.

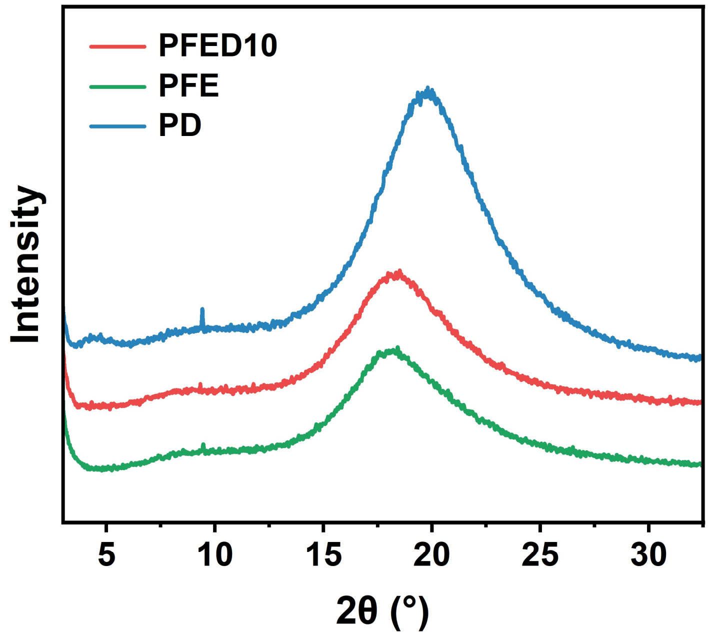

Supplementary Fig. 18. WAXD data for PFED10, PFE, and PD.

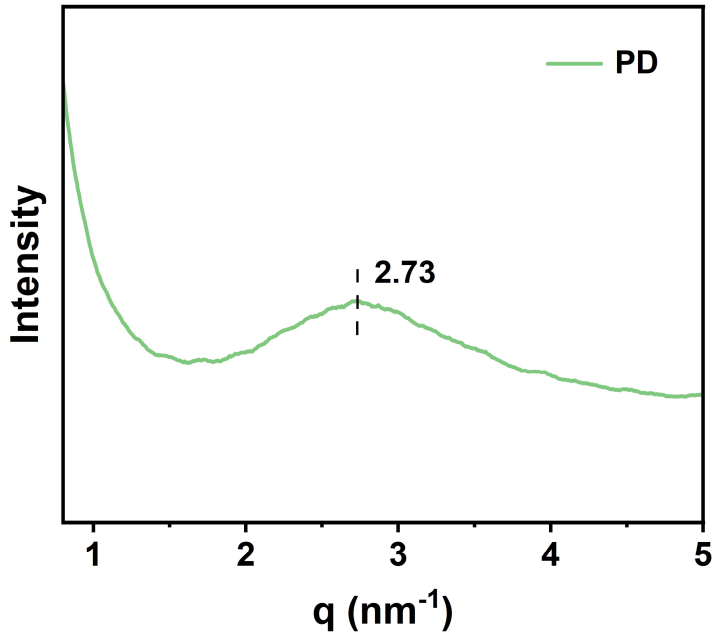

Supplementary Fig. 19. SAXD patterns of PD. The peak of PD indicates that the long side chains can form aggregation structures on the nanoscale.

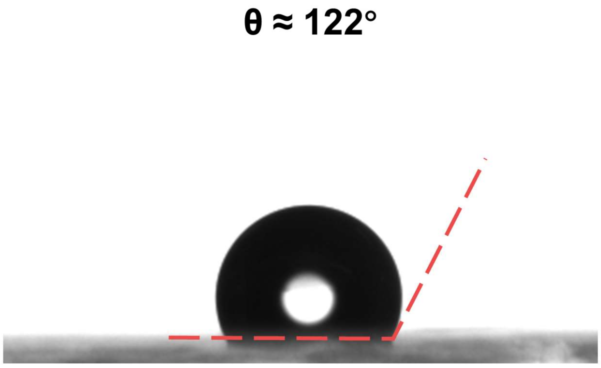

Supplementary Fig. 20. Water contact angle of the PFED10 film.

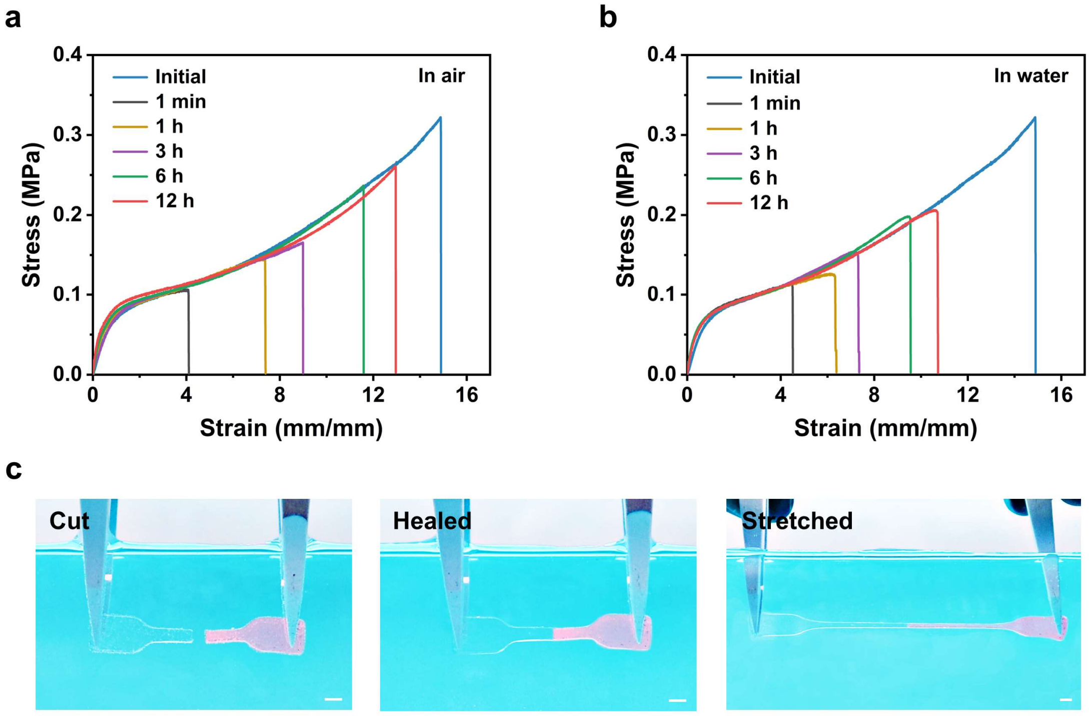

Supplementary Fig. 21. Self-healing properties of PFED10. a, Stress-strain curves of PFED10 samples after healing in the air for different times. **b**, Stress-strain curves of PFED10 samples after healing in water for different times. **c**, Photos of the cut samples healed underwater and stretched after healing for 1 min. Scale bar, 3 mm.

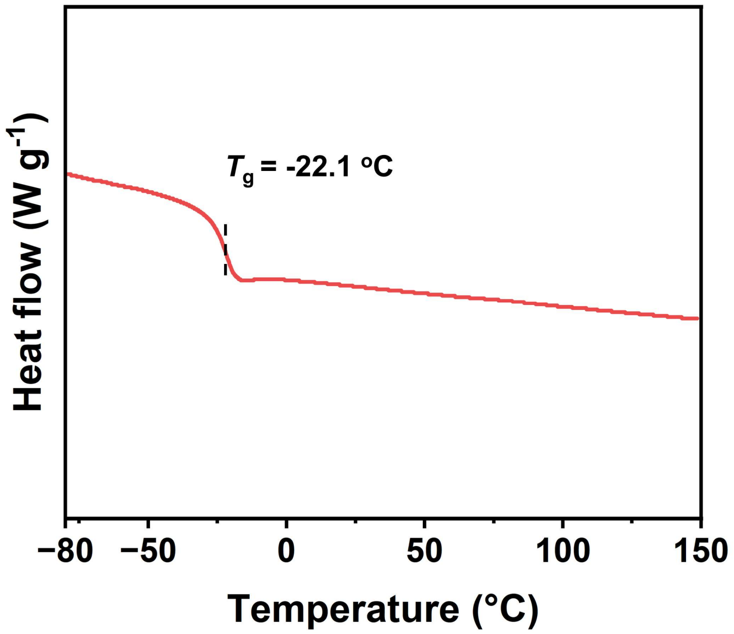

Supplementary Fig. 22. DSC curve of PFED10.

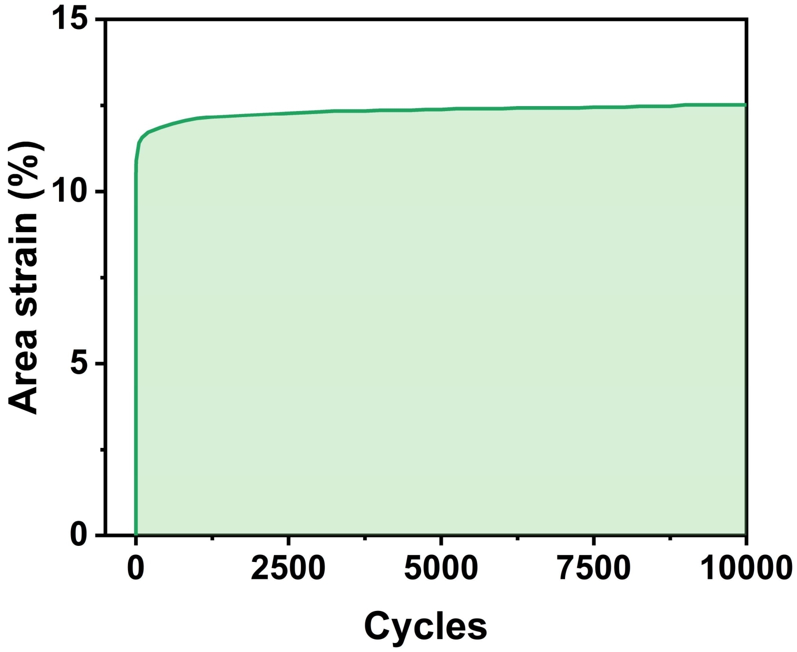

Supplementary Fig. 23. Cyclic actuation of PFED10 at 10.8 MV m^{-1} and 1 Hz for 10000 cycles without pre-stretch.

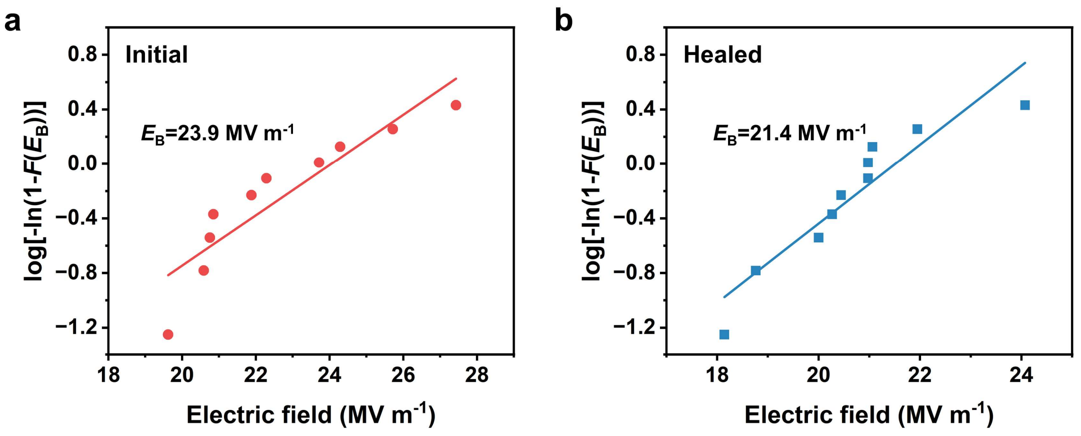

Supplementary Fig. 24. Electrical breakdown strength of PEFD10 before and after self-healing in water for 3 h without pre-stretch. a, The electrical breakdown strength of initial PFED10 samples without pre-stretch. **b**, The electrical breakdown strength of PFED10 samples after self-healing in water for 3 h without pre-stretch. The electrical breakdown strength of initial PFED10 samples and healed PFED10 samples are 23.9 MV m^{-1} and 21.4 MV m^{-1}, respectively. The electrical breakdown strength was maintained at about 89% of the initial value after self-healing.

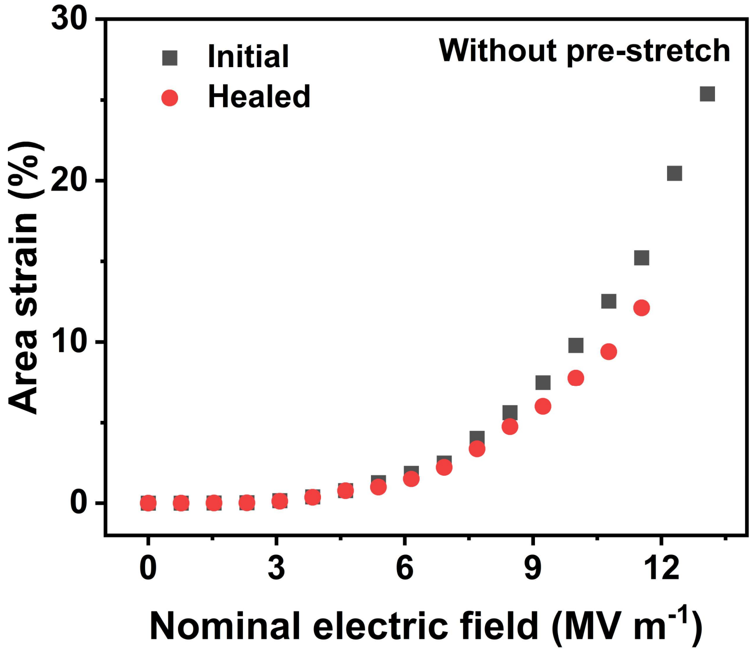

Supplementary Fig. 25. The actuation area strains of PFED10 before and after self-healing in water for 3 h without pre-stretch.

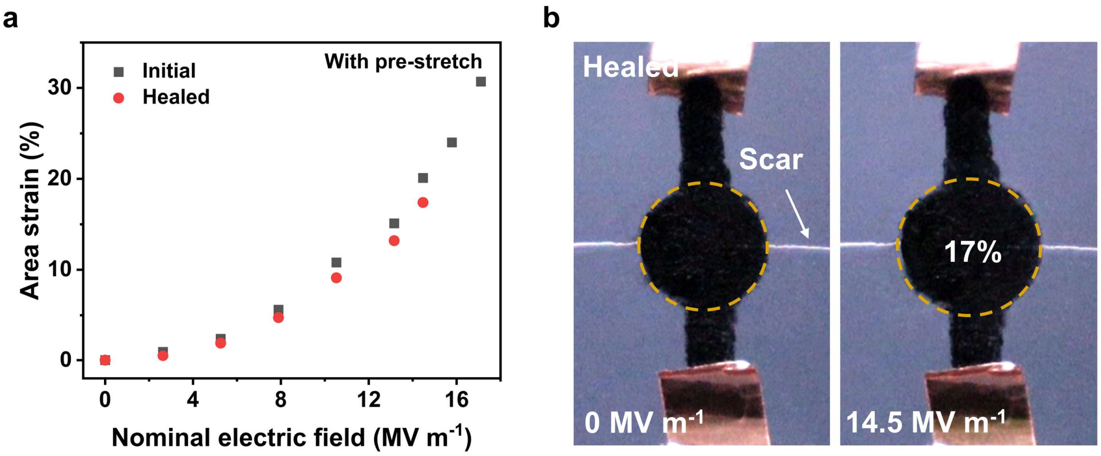

Supplementary Fig. 26. Self-healing property of DEA based on PFED10. a, The actuation area strains of PFED10 before and after self-healing in water for 3 h with 125% biaxial pre-stretch. **b**, Photographs of the actuation area strain of PFED10 after self-healing in water for 3 h at 14.5 MV m^{-1}.

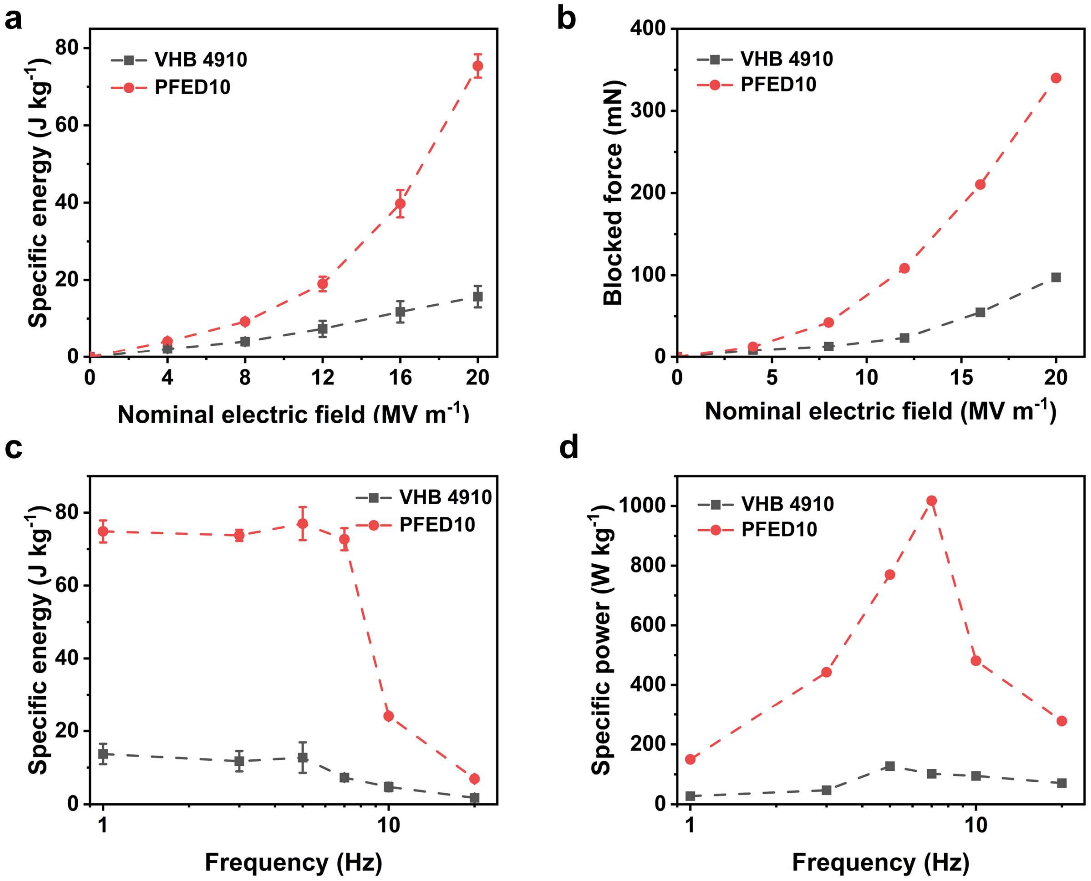

Supplementary Fig. 27. Specific energy and Specific power of VHB 4910 and PFED10 with a 60 g load. a, Specific energy of VHB 4910 and PFED10 films at different electric fields. The frequency was fixed at 0.5 Hz. Error bars show s.d., $n = 3$. **b**, Block force of VHB 4910 and PFED10 films at different electric fields with a 60 g load. **c**, Specific energy of VHB 4910 and PFED10 films at different frequencies. The driving electric field was fixed at 20 MV m^{-1}. Error bars show s.d., $n = 3$. **d**, Average specific power of VHB 4910 and PFED10 films at different frequencies.

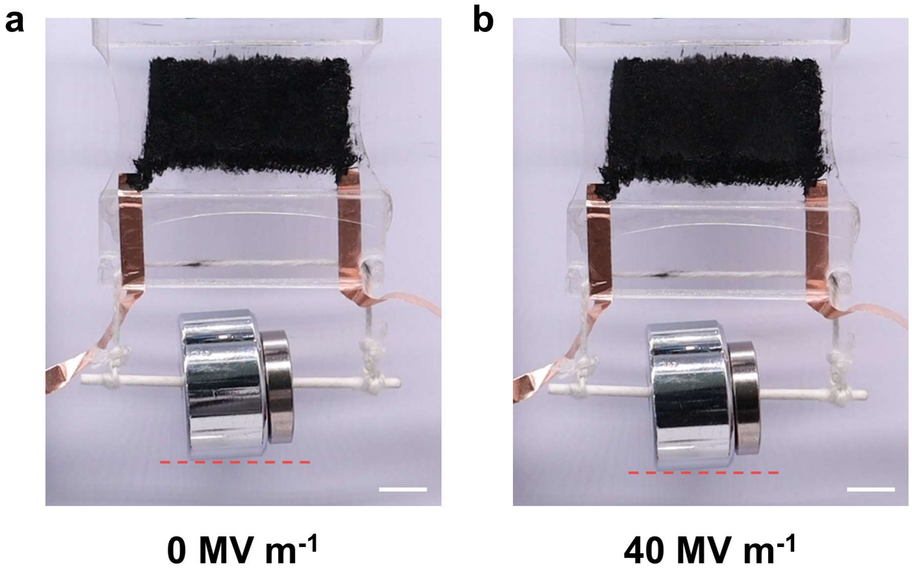

Supplementary Fig. 28. The linear actuation of VHB 4910 pure-shear DEA at 40 MV m^{-1} with a 120 g load. a, Voltage off. **b**, Voltage on. VHB 4910 achieved 16% linear strain and exhibited a maximum specific energy of 71 J kg^{-1} with a load of 120 g at 40 MV m^{-1} and 0.5 Hz. Scale bar, 1cm.

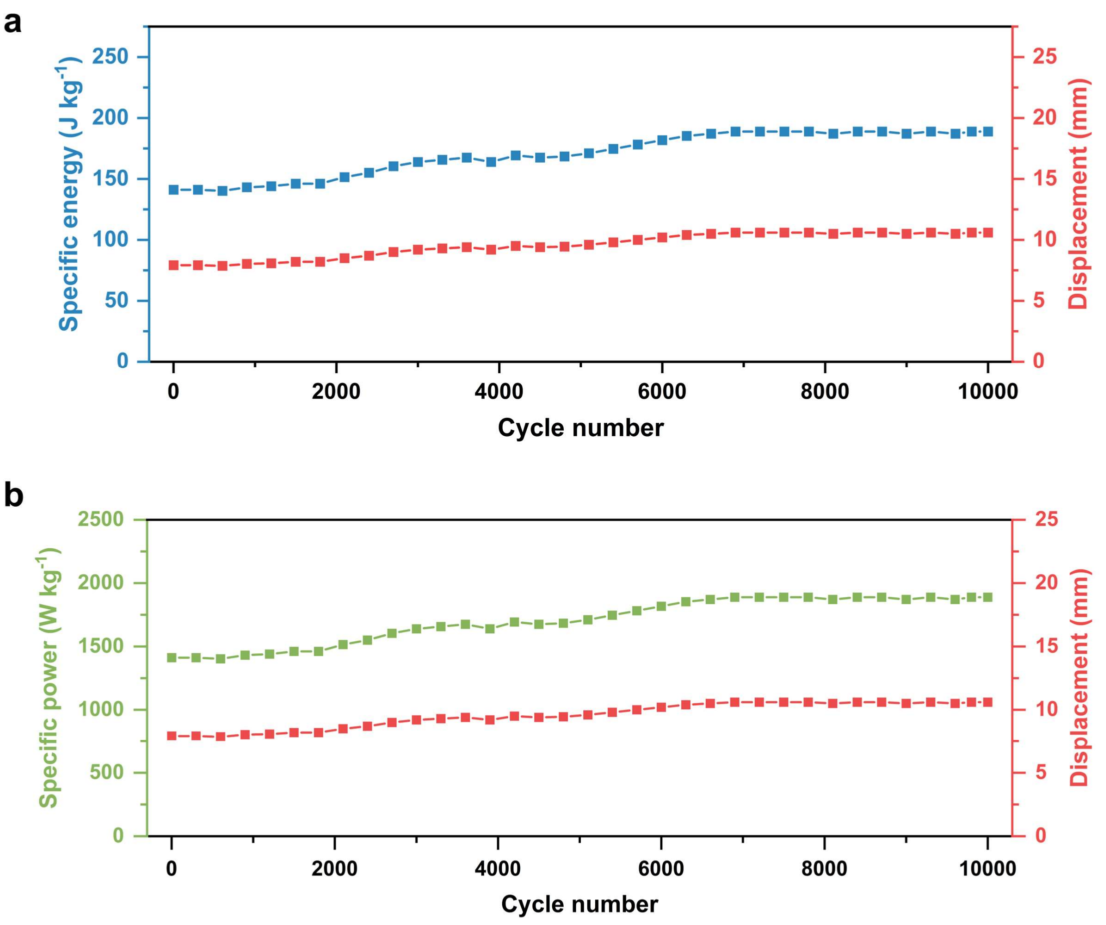

Supplementary Fig. 29. Specific energy and Specific power of PFED10 film in 10000 cycles actuation test with a 100 g load. a, Specific energy and linear displacement of the PFED10 film in 10000 cycles actuation test with a 100 g load. **b**, Specific power and linear displacement of the PFED10 film in 10000 cycles actuation test with a 100 g load. The electric field was 28 MV m^{-1} and the frequency was 5 Hz.

a **b**

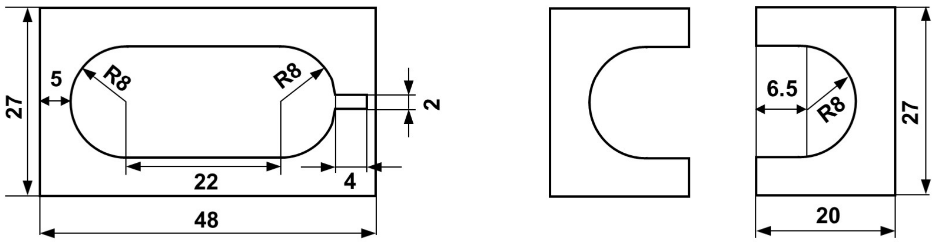

Supplementary Fig. 30. Geometric parameters of the soft robot body. a, Geometry dimension of the 0.1 mm thick PET frame. **b**, Geometry dimension of the 0.2 mm thick PET frame.

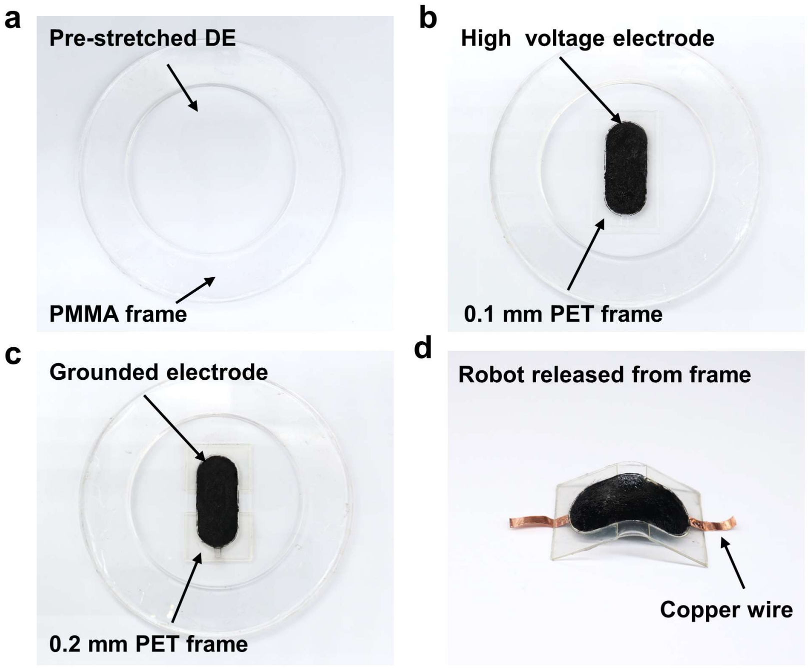

Supplementary Fig. 31. The fabrication process of the soft robot body. a, Pre-stretched DE membrane with PMMA frames to fix. **b**, High voltage carbon grease electrode and a 0.1 mm thick PET frame were stacked on one side of the DE membrane. **c**, Grounded carbon grease electrode and a 0.2 mm thick PET frame were stacked on the other side of the DE membrane. **d**, Releasing the soft robot body from the membrane and connecting the electrodes to a high-voltage source by copper wires.

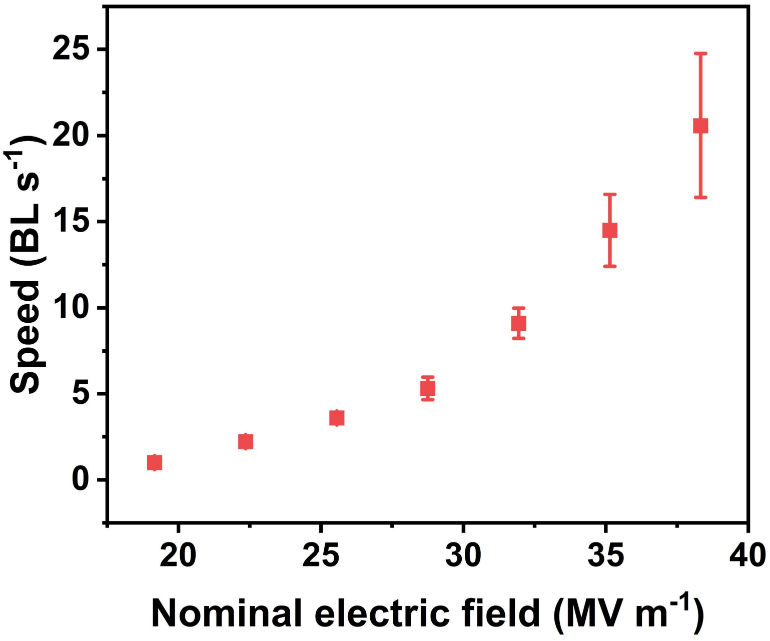

Supplementary Fig. 32. Running speeds of the soft robot based on PFED10 as a function of electric field at a fixed frequency of 30 Hz. Error bars show s.d., $n = 3$.

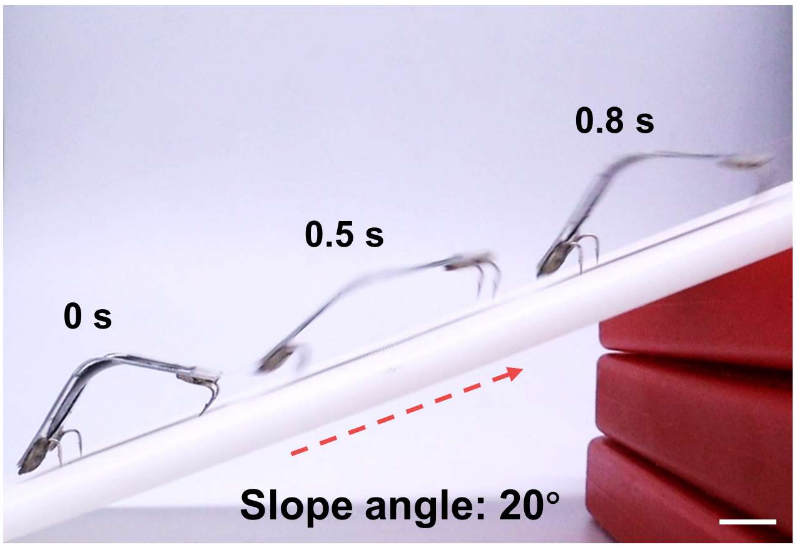

Supplementary Fig. 33. The soft robot based on PFED10 climbs a slope of 20° with a speed of 3.5 BL s^{-1}. Scale bar, 1 cm.

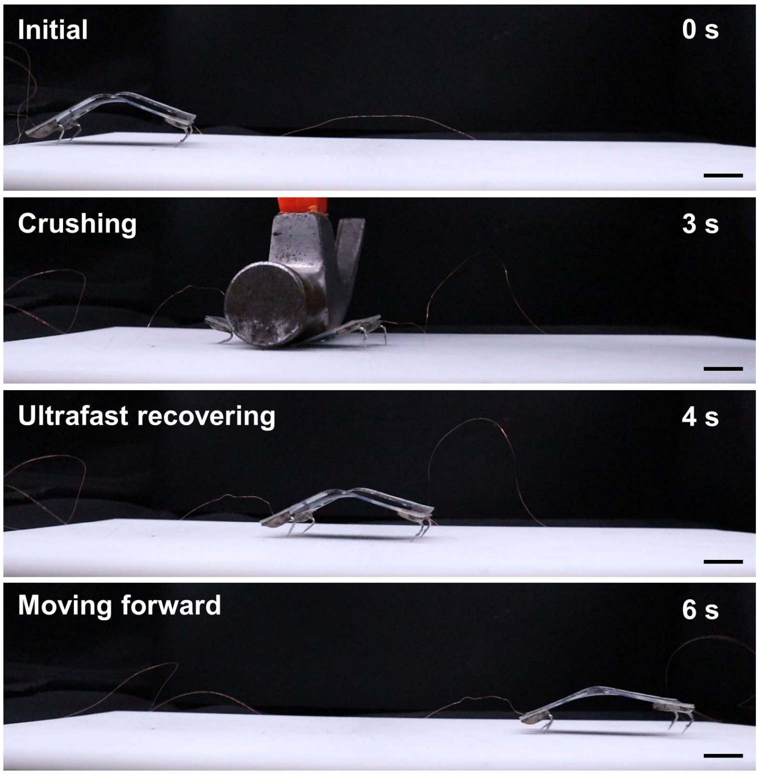

Supplementary Fig. 34. Robustness demonstration of the soft robot based on PFED10. A 1000 times heavier punch falling on the soft robot shows that the robot has the ability of quick recovery to make normal forward movements. Scale bar, 1 cm.

Supplementary Table 1. Formulation (molar ratio) and nomenclature of different DE films.

Samples	HFBA	EA	DA	HMPP
PF	100	0	0	0.5
PE	0	100	0	0.5
PD	0	0	100	0.5
PFE	100	10	0	0.55
PFED5	100	10	5	0.575
PFED10	100	10	10	0.6
PFED20	100	10	20	0.65

Supplementary Table 2. Electromechanical sensitivity of this work and other DEs.

Elastomer	Dielectric constant	Young's modulus (MPa)	Electromechanical sensitivity (MPa^{-1})	References
E3-Cl-20	5.4	0.3	18	48
C0-P0	5.3	0.2	26.5	49
BmimSbF6	7.61	0.15	50.73	50
E-CL2	18	0.35	51.4	51
C2	10.1	0.154	65.6	22
Cl-8.5	5.2	0.07	74.3	52
BAC2	5.75	0.073	78.8	19
ECN	17.5	0.155	112.9	53
PFED10	10.23	0.09	114	This work
