# Supplementary material for: A large-strain and ultrahigh energy density dielectric elastomer for fast moving soft robot
Source: Nat Commun. 2024 May 18;15:4222. doi: 10.1038/s41467-024-48243-y (PMC11102557; doi:10.1038/s41467-024-48243-y)
Supplement: Supplementary file 3 — Description of Additional Supplementary Files [file 41467_2024_48243_MOESM3_ESM.pdf]

Description of Additional Supplementary Files

Supplementary Movie Legends:

Supplementary Movie 1

Large actuation area strain of PFED10. This movie shows that PFED10 with 275% biaxial pre-stretch achieves a large actuation area strain at 46 MV m^{-1} and 0.1 Hz.

Supplementary Movie 2

Linear actuation of PFED10 pure-shear DEA at different frequencies with a 120 g load. This movie shows that PFED10 pure-shear DEA achieves different linear actuations from 1 Hz to 20 Hz under a load of 120 g demonstrating ultrahigh energy density and excellent power density. The nominal driving electric field is 40 MV m^{-1}. The active area of DE film is about 4 cm long and 2.5 cm wide.

Supplementary Movie 3

PFED10 pure-shear DEAs lift a 1.5 kg bucket. This movie shows that two large PFED10 pure-shear DEAs, which can deliver a large actuation force, are able to lift a 1.5 kg bucket at 35 MV m^{-1} and 2.4 Hz. The active area of DE film is about 6 cm long and 5.3 cm wide.

Supplementary Movie 4

Ultrafast moving soft robot based on PFED10. This movie shows that the soft robot prepared by PFED10 runs on a sawtooth-shaped substrate with a maximum speed of 20.6 BL s^{-1} at 38 MV m^{-1} and 30 Hz, demonstrating the fastest speed among DEA-driven soft robots. We slowed the playback down 10 times to track the running robot better.

Supplementary Movie 5

Running of PFED10-based soft robots on different substrates. This movie shows that the soft robots prepared by PFED10 can fast run on different substrates including

sandpaper-p600, silicone, paper, plastic, and glass at 38 MV m^{-1} and 30 Hz.

Supplementary Movie 6

Load-carrying test of soft robot based on PFED10. This movie shows that the soft robot prepared by PFED10 carries a load of about 17 times its own weight at 35 MV m^{-1} and 10 Hz, demonstrating an excellent load-carrying capacity.

Supplementary Movie 7

Slope-climbing tests of soft robot based on PFED10. This movie shows that the soft robot prepared by PFED10 climbs the slopes of 20 degrees and 45 degrees at 35 MV m^{-1} and 10 Hz, demonstrating a good climbing capacity.

Supplementary Movie 8

Low-voltage driven soft robot based on PFED10. This movie shows that the soft robot prepared by 26 μm PFED10 film can be driven at 400 V and 10 Hz, demonstrating an excellent low-voltage driven performance of PFED10. The scale factor of the high-voltage source is 1000.
